# Extraordinary diversity of telomeres, telomerase RNAs and their template regions in Saccharomycetaceae

**DOI:** 10.1038/s41598-021-92126-x

**Published:** 2021-06-17

**Authors:** Vratislav Peska, Petr Fajkus, Michal Bubeník, Václav Brázda, Natália Bohálová, Vojtěch Dvořáček, Jiří Fajkus, Sònia Garcia

**Affiliations:** 1 Institute of Biophysics, Academy of Sciences of the Czech Republic, Brno, 61265 Czech Republic; 2 Department of Experimental Biology, Faculty of Science, Masaryk University, Brno, 62500 Czech Republic; 3 Mendel Centre for Plant Genomics and Proteomics, CEITEC, Masaryk University, Brno, 62500 Czech Republic; 4 Institut Botànic de Barcelona (IBB-CSIC, Ajuntament de Barcelona), Passeig del Migdia s/n, 08038 Barcelona, Catalonia Spain

**Keywords:** Computational biology and bioinformatics, Evolution, Molecular biology

## Abstract

Telomerase RNA (TR) carries the template for synthesis of telomere DNA and provides a scaffold for telomerase assembly. Fungal TRs are long and have been compared to higher eukaryotes, where they show considerable diversity within phylogenetically close groups. TRs of several Saccharomycetaceae were recently identified, however, many of these remained uncharacterised in the template region. Here we show that this is mainly due to high variability in telomere sequence. We predicted the telomere sequences using Tandem Repeats Finder and then we identified corresponding putative template regions in TR candidates. Remarkably long telomere units and the corresponding putative TRs were found in *Tetrapisispora* species. Notably, variable lengths of the annealing sequence of the template region (1–10 nt) were found. Consequently, species with the same telomere sequence may not harbour identical TR templates. Thus, TR sequence alone can be used to predict a template region and telomere sequence, but not to determine these exactly. A conserved feature of telomere sequences, tracts of adjacent Gs, led us to test the propensity of individual telomere sequences to form G4. The results show highly diverse values of G4-propensity, indicating the lack of ubiquitous conservation of this feature across Saccharomycetaceae.

## Introduction

Chromosomes are capped by nucleoprotein structures called telomeres, which are involved in several important functions such as the regulation of gene expression, recombination, mitosis and meiosis^1–4^. Telomeres protect chromosome termini from eliciting the DNA damage response and play a fundamental role in longevity and cell proliferation^5^. Most telomeres carry 3´ overhangs that can be generated by incomplete synthesis at the lagging strand after degradation of the RNA primer of the distal-most Okazaki fragment and resection, and/or by resection of a blunt-end telomere intermediate resulting from leading strand synthesis^6–8^. This end-replication problem is solved by an enzyme called telomerase^9^. While a deficit in telomerase would translate into shorter telomeres over each replication, triggering senescence, its continued or dysregulated expression could unbalance growth control, potentially leading to oncogenesis, if control mechanisms do not direct the cell e.g., to apoptosis^10^. Telomerase is responsible for telomere maintenance in most eukaryotes and had originated early in eukaryotic evolution in association with linearization of the chromosomes^11–14^. Telomerase consists of two main parts; a catalytic protein component—telomerase reverse transcriptase (TERT), and the telomerase RNA (TR) subunit. TR contains a template region directly responsible for encoding the telomere sequence, from which the enzyme repeatedly copies (via reverse transcription) a sequence motif that forms the tandem DNA repeat arrays of telomeres. Telomerase is not completely universal^15^, since, for example, in *Drosophila*, telomerase had been lost and telomeres composed of short tandem repeats were replaced with terminal retrotransposons that use targeted retrotransposition, thus engaging in a mechanism similar to reverse transcription^16,17^. Nevertheless, telomerase remains the most common mechanism to maintain telomeres across eukaryotes, and without any known exceptions among plants^14,18–20^.

**Figure 1:**
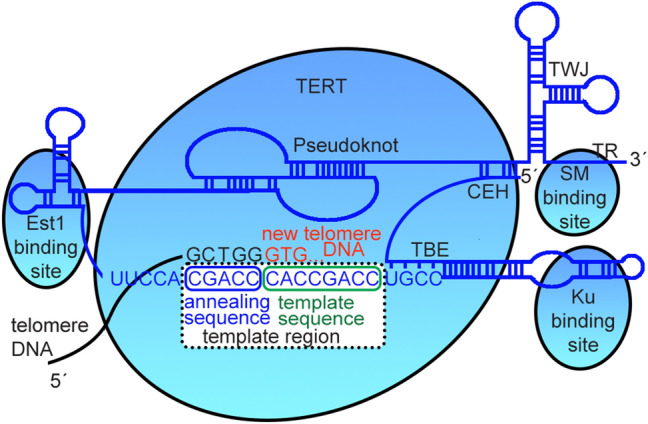
Telomerase RNA organization fo*r Lachancea* sp. The most relevant binding sites for protein-RNA interactions are indicated by blue ellipses (Est1, SM and KU), and the bigger one is the contact region with the reverse transcriptase (TERT). *TBE* template boundary element, *TWJ* three-way junction, *CEH* core enclosed helix. Adapted from Waldl et al.^36^ The template region is located between the EST1 binding site and Ku binding hairpins. (Figure created using Adobe Photoshop 22.2.0).

The distribution of nucleotides is uneven between and also along the telomere DNA strands as it is obvious from telomere motif consensus in many plants, vertebrates, insects, some fungi and many others—(T_x_A_y_G_z_)_n_. The yeast telomeric repeat sequence (TG_1–3_ where more than 80% is TGTGGG) was first described in *Saccharomyces cerevisiae* in two back-to-back papers in Nature in 1984^21,22^. Later works showed unprecedented variability in the telomere sequences in yeasts, and made the eukaryotic consensus obsolete. The step-by-step telomere sequence evolution in yeasts was currently proposed^23^. However, any obvious feature of the telomere sequence common for all yeasts is difficult to determine, except the clusters of at least two adjacent guanines in the telomere motifs, although the significance of it has not been yet clarified. Several hypotheses explain the role of this clustering e.g., in binding of conserved telomere proteins or forming specific secondary structures, such as G-quadruplexes (G4), which were assumed to occur in telomere regions of several species, including human and *S. cerevisiae*^24,25^. The asymmetric distribution of guanines and cytosines between the complementary strands arise from the fact that the template region of TR is C-rich and, consequently, the telomere DNA strand produced by the telomerase is G-rich. The template region is longer than the telomere repeat unit, which allows a complete repeat to be translocated to an annealing part of the template region for addition of the next repeat, according to the template sequence^19,26^ (Fig. 1). It has been shown in various plants that the same telomere sequence can originate from template regions with different lengths and annealing sequences. Moreover, in *Arabidopsis thaliana*, the annealing sequence can be as short as two nucleotides^27,28^.

The length of the whole telomerase RNA is highly variable across organisms, ranging from 150 to more than 2000 nucleotides^29,30^, with the human sequence of 451 nt^31^ or baker's yeast of 1158 nt^32,33^ as examples. The reasons for this diversity are unknown, although it may be related to structural rather than encoding functions of TRs. In the case of yeasts, several structural features were identified, such as the Ku binding hairpin, the Est1 binding site, the pseudoknot, the three-way junction and the SM binding site^34^ (Fig. 1). The template region is, in some cases, located between the Ku and the Est1 binding sites. The longest possible template region, which includes short direct repeats at both ends, can be easily identified if the telomere sequence and telomerase RNA for a species are available^35^. For example, in *Saccharomyces cerevisiae*, the real template is formed by 5′-CAccacacccacacACA^32^, while the predicted template region obtained in our approach (i.e., corresponding to the tandem repeats found by Tandem Repeats Finder) is narrower (in lowercase). Nevertheless, predicting promising TRs by using BLAST searches, as recently applied in land plants^27^, is a challenging task even among relatively closely related taxa. In the case of considerable evolutionary divergence among species, such as in yeasts, the BLAST strategy is not feasible to start with, given the low sequence conservation among TRs^36^. Although TRs are variable in the primary structure and are known only in a limited number of organisms, innovative bioinformatics tools using phylogenetics and covariance models^33,37^ and conserved secondary structures in TRs^38–40^ have led to discoveries even in more distant eukaryotic kingdoms such as protostomes^41^.

In yeasts, TR sequences are particularly diverse in length and complexity, and only a few are available. A recent survey aimed to provide yeast TR sequences, identifying 46 cases, from which 27 telomerase RNAs were found for the first time in the family Saccharomycetaceae. Several strategies were used, including the comparison of common sequence features of known TRs in this family, synteny-based homology searches, the use of known telomere template sequences, or employing a set of known TR regions for covariance model-based searches and BLAST queries (including all template regions available from Saccharomycetaceae at that time)^36^. However, these authors failed to find template regions in 13 of the TRs that they attempted to analyse, mostly due to the lack of knowledge of specific telomere sequences. Here we complement and expand these results by predicting telomerase RNAs in several closely related species, describing their telomere motifs and predicted template regions. Interestingly, we predict one of the longest known telomere units across eukaryotes (28 bp) in the genus *Tetrapisispora*, as well as its corresponding TR, including the template region. We also compare propensities of the identified telomere candidates to form a guanine quadruplex (G4; G-quadruplex), and finally, we discuss the significance of motif length in relation to telomerase processivity.

## Results

### Validation of the approach

**Figure 2:**
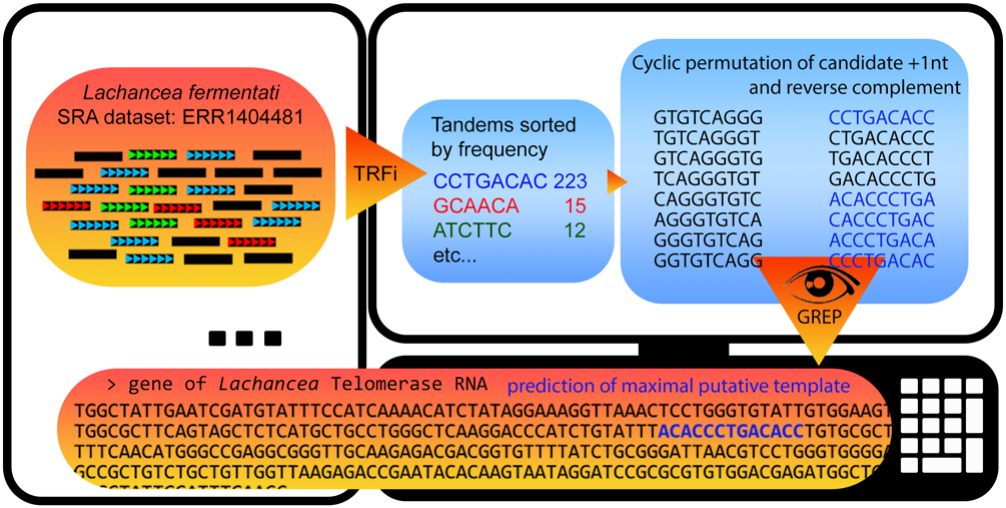
Example of the pipeline for finding telomere motifs and predicting template regions in *Lachancea fermentati*. NGS data in short reads were processed by Tandem Repeats Finder (TRFi) to obtain a list of tandem motifs sorted according to their frequency in the dataset (indicated by different colours). Permuted sequences and their reverse complements were checked using a full text search based on the grep command in a Linux terminal for their presence between the Ku binding hairpin and the Est1 binding site. (Figure created using Adobe Photoshop 22.2.0).

We analysed 38 genomic datasets from Saccharomycetaceae. These datasets represented species from the genera *Candida*,* Kluyveromyces*, *Lachancea*, *Saccharomyces*,* Tetrapisispora*, *Torulaspora* and *Zygosaccharomyces*. We successfully validated our approach on the raw data from SRA database (NCBI) in 19 species from the first four genera (Supplementary Table S1). Therefore, we omitted trimming and prefiltering as the downloaded data exhibited sufficient quality for the prediction of telomere sequence as one of the most abundant tandems. Thus, we neglected a little probability that low-quality reads can produce specific telomere candidates with regular pattern corresponding to the telomerase RNA counterparts. The in silico approach we used in this work is summarized in Fig. 2. Briefly, the telomere candidates were usually among the most prevalent tandems from Tandem Repeats Finder analysis (TRFi) and showed conserved clusters of two or more adjacent guanines (or cytosines in reverse complement in genomic data). TRFi is a reference free alignment-based algorithm which can be set by several parameters, including score for matches, mismatches, and gaps^42^, more details about our setup are described in Methods. We use (for simplicity) the term “maximum putative template” for the longest possible region from TR that corresponds to any (mostly only one) tandem repeat consensus obtained in TRFi. It does not mean that we delimit the maximum for a real template. Rather, the maximum putative template always forms at least a substantial core part of the experimentally verified template region. Our predictions of basic telomere repeats and maximum putative template regions were not identical, but consistent with the repeats and template regions identified previously in biochemical experiments^29,32–36,43–47^. Thus, for most of them, we put the new coordinates of the maximum putative template region according to the consensus telomere from TRFi results in the Supplementary Table S2. In all cases, the telomere sequence candidate from TRFi shared 100% homology with the maximum putative template regions.

### Complete predictions

We have predicted new TRs, their template regions, and telomere sequences in the species *Lachancea cidri*, three species of *Tetrapisispora* (*T. fleetii*, *T. iriomotensis*, and *T. namnaoensis*), three species of *Torulaspora* (*T. franciscae*, *T. maleeae* and *T. pretoriensis*), and *Zygosaccharomyces sapae*. These results were based on a combination of the outcome of synteny-based homology search for telomerase RNA, and the TRFi analysis.

### Additional predictions

We identified telomere candidates and template regions in four species and one strain of *Lachancea* (*L. fermentati*, *L. meyersii*, *L. mirantina*, *L. nothofagi* and *L.* sp. CBS 6924), two species of *Torulaspora* (*T. delbrueckii* and *T. microellipsoides*) and two species of *Zygosaccharomyces* (*Z. bailii* and *Z. rouxii*) (Supplementary Table S1).

### Modified TRFi in case of *K. lactis*, *L. kluyveri*, and *T. blattae*

A modified setting of Tandem Repeats Finder parameters with final reduction in number from five to two units of detected motifs of at least 20 nt in tandem uncovered the proper telomere sequence in *L. kluyveri* but not in *K. lactis*. For the latter, we were not able to detect the telomere sequences in the available datasets despite the fact that the motif (25 nt), TR, and template region had been reported previously^34,36,43^. In comparison, the reads of *L. kluyveri* (100 nt) were not long enough to detect at least five adjacent tandem repeats, which illustrated how this parameter could act as a bottleneck of the method. If we set this parameter to a lower stringency e.g., to two adjacent repeats, then we should be able to detect the telomere sequence such as in *L. kluyveri* and *T. blattae* (Supplementary Table S2). Nevertheless, setting the default parameter to five makes the method selective for telomere repeats that are usually of very low complexity as compared to e.g., short interstitial telomere sequences type II and III^48^ and teloboxes present in promoters^49^, where less than five regular motifs are expected. On the other hand, the dataset analysed from *K. lactis* was produced on the HiSeq X Ten platform, which systemically under-represents (and in some cases completely misses) telomere sequences, as reported previously^50^.

**Figure 3:**
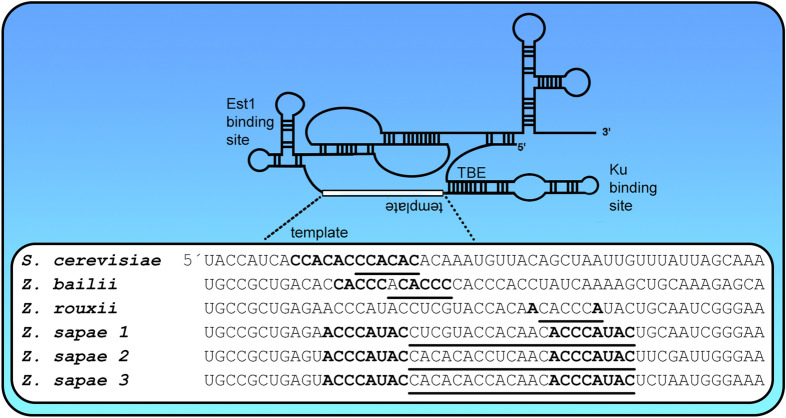
Schematic picture of telomerase RNA and template regions in *Saccharomyces cerevisiae*, *Zygosaccharomyces bailii*, *Z. rouxii*,* and Z. sapae*. We identified the same putative consensus telomere sequence as 5′-ACACCC (or its permutations) in *Saccharomyces cerevisiae*,* Zygosaccharomyces bailii* and *Z. rouxii*, although the template regions, annealing and telomere synthesis during reverse transcription differed in all three species. The corresponding template regions are indicated in bold in the sequences (direct repeats), and underlined fonts (template sequence), respectively. (1) In *S. cerevisiae* the maximum template region consists of two adjacent CCACAC repeats. (2) In *Z. bailii* the template region is formed by a shorter sequence than in *S. cerevisiae* and enables the annealing of five nucleotides and the addition of six nucleotides complementary to ACACCC. (3) The template region in *Z. rouxii* is a bit paradoxical. The synthesis is probably running on the template sequence CACCCA (a permutated version of ACACCC) and annealing occurs through a single base pair (the first A in the template). However, the template region from *Z. rouxii* corresponds exactly to a much longer telomere sequence (21 nt) from the species *Z. sapae*, with a potentially longer annealing (up to 8 bp). Three TR paralogs were found in *Z. sapae.* (Figure created using Adobe Photoshop 22.2.0).

All predictions and validations are summarized in Supplementary Table S1. The details and results from TRFi are shown in Supplementary Table S2. Several additional key results are: **(1)** long telomere motifs were found in *Kluyveromyces* sp. (25 nt) and *L. kluyveri* (26 nt); **(2)** *L. cidri* and *L. fermentati* exhibited the telomere motif, (CTGACACC)n. Their candidate TRs shared 56% identity and the maximum putative template was identical for both of them. **(3)** *L. mirantina*, *L. waltii*, and *L. thermotolerans* shared the telomere motif (CCAACACC)n, but their template regions differed. Similarly, **(4)** some *Lachancea* sp. analysed shared the (CACCCAGC)n but their template regions differed. **(5)** We detected short (6 nt—the same as in *S. cerevisiae*) and long (21 nt) telomere motifs in *Zygosaccharomyces*. Remarkably, the annealing part of the template region in *Z. rouxii* was formed by a single nucleotide. *Z. sapae* possessed three TR paralogs with distinct template regions (Fig. 3, Supplementary Table S1 and Supplementary Table S2). **(6)** The telomere motif in *T. microellipsoides* was 8 nt long, and the rest of the genus showed motifs of 13 nt. **(7)** In the *Tetrapisispora* sp. analysed two telomere motifs per species were detected (Supplementary Table S1).

### Prediction of G4 propensity in telomere sequences

We analysed the set of 20 consensus sequences identified by Tandem Repeats Finder (Supplementary Table S3) for the presence of G4 forming sequences (GFS). The predicted telomere sequences varied in the length of their repeat units (6 to 28 nt) and in GC content (34.5–69.1%). The QGRS mapper identified 19 out of the 20 sequences as potential GFS with an average score of 27 (from 8 to 63). The G4Hunter score was found to be above the threshold of 1.2 in only six sequences. The highest G4Hunter score was 2.2 for the telomere sequences from *L. mirantina*, *L. thermotolerans* and *L. waltii* (TGTTGGGG), followed by the sequence repeat from *C. glabrata* (GTCTGGGTGCTGTGGGGTC) with a G4Hunter score of 1.84. Species from the *Kluyveromyces* genus with scores under 0.6 represented the other extreme of the scale. The simulated sequence from the *Saccharomyces* species tested, *Z. bailii*, and *Z. rouxii*, (TGTGGG), was of G4Hunter score 1.72. These G4Hunter scores were comparable to those of real telomeres from *S. cerevisiae*, which varied from 1.56 to 1.68. For more details and a comparison between the QGRS versus G4Hunter results, see Supplementary Table S3.

### In vitro validation of G4 formation

**Figure 4:**
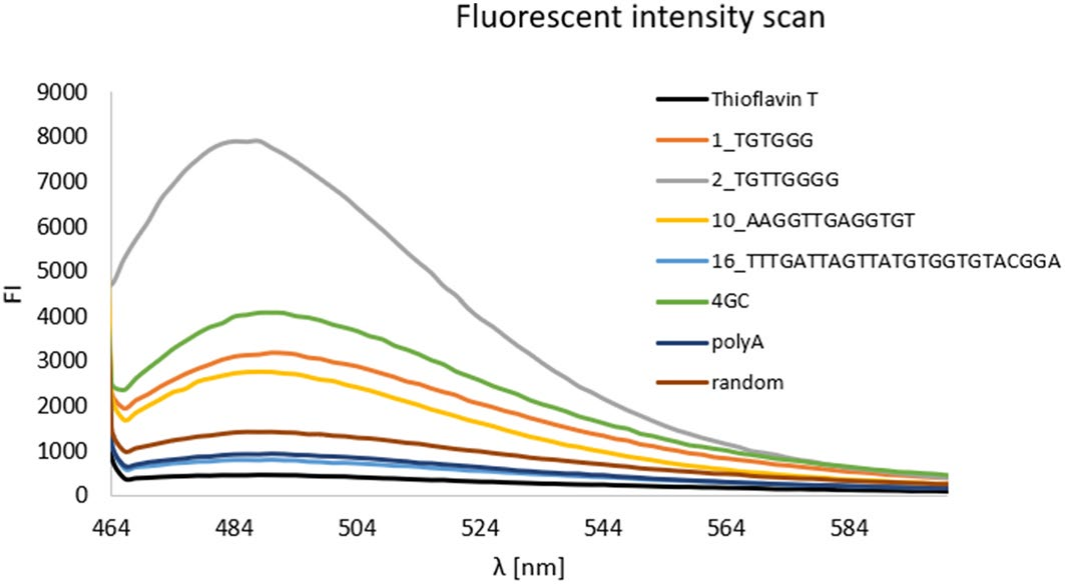
Thioflavin T (ThT) fluorescence assay. We tested four oligonucleotides representing selected telomere sequences from *Sachcaromyces* sp., *Zygosaccharomyces* sp., *Lachancea* sp., *Torulaspora* sp., and *Kluyveromyces marxianus*; three controls—one G4-forming sequence (called 4GC) and two negative controls (polyA and random). The G4 propensity was confirmed in vitro by both methods in three out of four telomere sequences tested as well as in positive control (in this figure, Supplementary table S4). The only sample sequence which did not form G4 in vitro was the one that was negative by prediction also. (Figure created using Adobe Photoshop 22.2.0).

The predicted propensity of selected telomeric sequences to form G4 was experimentally tested by a combination of two methods, circular dichroism (CD) spectroscopy, and the Thioflavin T (ThT) fluorescence assay (Supplementary Table S4) to assess G4 formation. Taken together these methods provide more reliable answer than any of them separately^51^. We tested four oligonucleotides representing selected telomere sequences from *Sachcaromyces* sp., *Zygosaccharomyces* sp., *Lachancea* sp., *Torulaspora* sp., and *Kluyveromyces marxianus*; three controls—one G4-forming sequence (called 4GC) and two negative controls (polyA and random). The G4 formation was confirmed by both methods in three out of four telomere sequences tested as well as in the positive control (Fig. 4, Supplementary table S4). The only sample sequence which did not form G4 in vitro was the one that was also predicted to be negative. As evidence of G4 formation in vitro, we considered the shift of the CD spectroscopy peak to the maximum typical for the particular type of G4 (parallel G4 = maximum ≈ 264 nm, minimum ≈ 245 nm, antiparallel G4 = maximum ≈ 295 nm, minimum ≈ 260 nm, hybrid = maximum ≈ 295 and 260 nm, minimum ≈ 245 nm) and a stronger signal when G4 was stabilized in the presence of K+ ions. ThT is a light-up probe with an increased fluorescence intensity specific for G4 detection in vitro (Fig. 4, Supplementary Table S4).

## Discussion

The approach used here combines a prediction of telomere sequence in silico and its support by congruence with the maximum putative template region in independently identified TR candidates. Our findings are consistent with those previously reported in 18 cases, including the already published data from species of the genera *Candida*, *Kluyveromyces*, *Lachancea* and *Saccharomyces*^33–36,43,45,47,52^.

The consensus telomere sequence detected in all *Saccharomyces* analysed was predominantly (CCACAC)n, already described as the basic telomere motif in *S. cerevisiae*^21,43,53^. All species tested showed congruence between our telomere sequence prediction from genomic data, and prediction of the template region from previous reports^34,36,43^. We also detected some other motifs that could represent telomere sequence variability (telomerase slippage, abortive synthesis, and sequence degeneration) but they were not considered representative (Supplementary Table S2). *Candida glabrata*’s relatively long telomere motif (16 nt) and its corresponding template region was also detected, although the relative length of the template region with respect to the telomere motif was rather short (1.19)^29^. Other telomere-like sequences were found for this species at an even higher frequency (Supplementary Table S2), although they did not correspond to either the published template regions or our maximum putative template region.

Considerable variability in telomere units was described at the level of genome assembly in numerous species of Saccharomycetaceae, e.g., *T. iriomotensis*, *S. cerevisiae*, and *Kazachstania exigua*^23^. We show here that even telomeres with complex sequences have a clear consensus with its maximum putative template counterpart. We have predicted telomere sequences for 18 species, with up to three possible variants in some of them (e.g., *Z. sapae*). In 10 of these, an approach based on the analysis of predicted TRs, without knowing the telomere sequence, had failed to recover the template region^36^. Here, we report, for the first time, the telomere sequence and the maximum putative template regions in *Toluraspora*, using the previously predicted TRs from *T. delbrueckii* and *T. microellipsoides* and our own TR predictions for other species of *Torulaspora*. Our results from Saccharomycetaceae also support some of the putative telomere sequences in comprehensive telomere searches across Ascomycota^23^. *Torulaspora delbrueckii* and *T. microellipsoides* are examples of the intrageneric variability of the telomere sequence. Several telomere-like motifs were also detected in *T. delbrueckii* (Supplementary Table S2), although they did not match the template region and their frequency was much lower. They may represent, e.g., variability in the telomere sequence or remnants of ancestral motifs. The analysis of genomic data from three additional *Torulaspora* species (*T. franciscae*, *T. maleeae* and *T. pretoriensis*) showed that they presented the same telomere sequence candidate as *T. delbrueckii*, however the template region of *T. maleeae* was shorter by 6 nucleotides and annealing was only possible with 2 nucleotides. This illustrates that even the same telomere motif can be encoded by a dramatically different template region (see Supplementary Table S1).

As with *Torulaspora*, Waldl et al.^36^ predicted TRs in *Zygosaccharomyces bailii* and *Z. rouxii*, but they did not show template regions in any of these sequences. We reanalysed these species datasets, and one more from the same genus (*Z. sapae*), with heterogeneous results. In the case of *Z. bailii* and *Z. rouxii*, we predicted CCACAC as a telomere sequence candidate and the corresponding template regions in TRs. We obtained an unexpected outcome however, for *Z. sapae*. First of all, we found three homologs of TR, each with a unique template region in this species and its counterpart in the TRFi output. Second, the three telomere motifs were relatively long (21 nt) and their frequency in the dataset differed by orders of magnitude (from 7.2 × 10^–6^ to 3.0 × 10^–5^). Third, one of the *Z. sapae* template regions overlapped with the template in *Z. rouxii*, which, paradoxically, had a much shorter prevalent telomere motif, 6 instead of 21 nt. The paradox of a short telomere motif (CACCCA)n in *Z. rouxii* and a long telomere candidate from *Z. sapae* (CCCATACCTCGTACCACAACA)n may have a simple explanation. It was shown that the base pairing between the template RNA and the telomere DNA is limited not only by the structure of the telomerase RNA but also by the protein subunit TERT^54^. Thus, we speculate that the two species may differ in their prevalent telomere sequences despite having the same template and surrounding regions because the change is caused by the preferred annealing mode. This explanation was also recently proposed for the transition between plant- and human-type telomere sequences in marine plants from the *Zostera* genus^28^. In summary, *Z. sapae* is an extreme example of intraspecific telomere sequence variability ensured by multiple TR paralogs across all eukaryotes.

We further analysed the *Lachancea* species, which also illustrate telomere sequence variability at the genus level, both in nucleotide composition and in sequence length (from 8 to 26 nt). We described new telomere motifs for 6 taxa (Supplementary Table S1) and new TR, including the template region, in *L. cidri*. We found possible signs of telomerase errors in *L. thermotolerans* (and in *L. cidri* to a lesser extent), in which several similar motifs were proposed (Supplementary Table S2).

**Figure 5:**
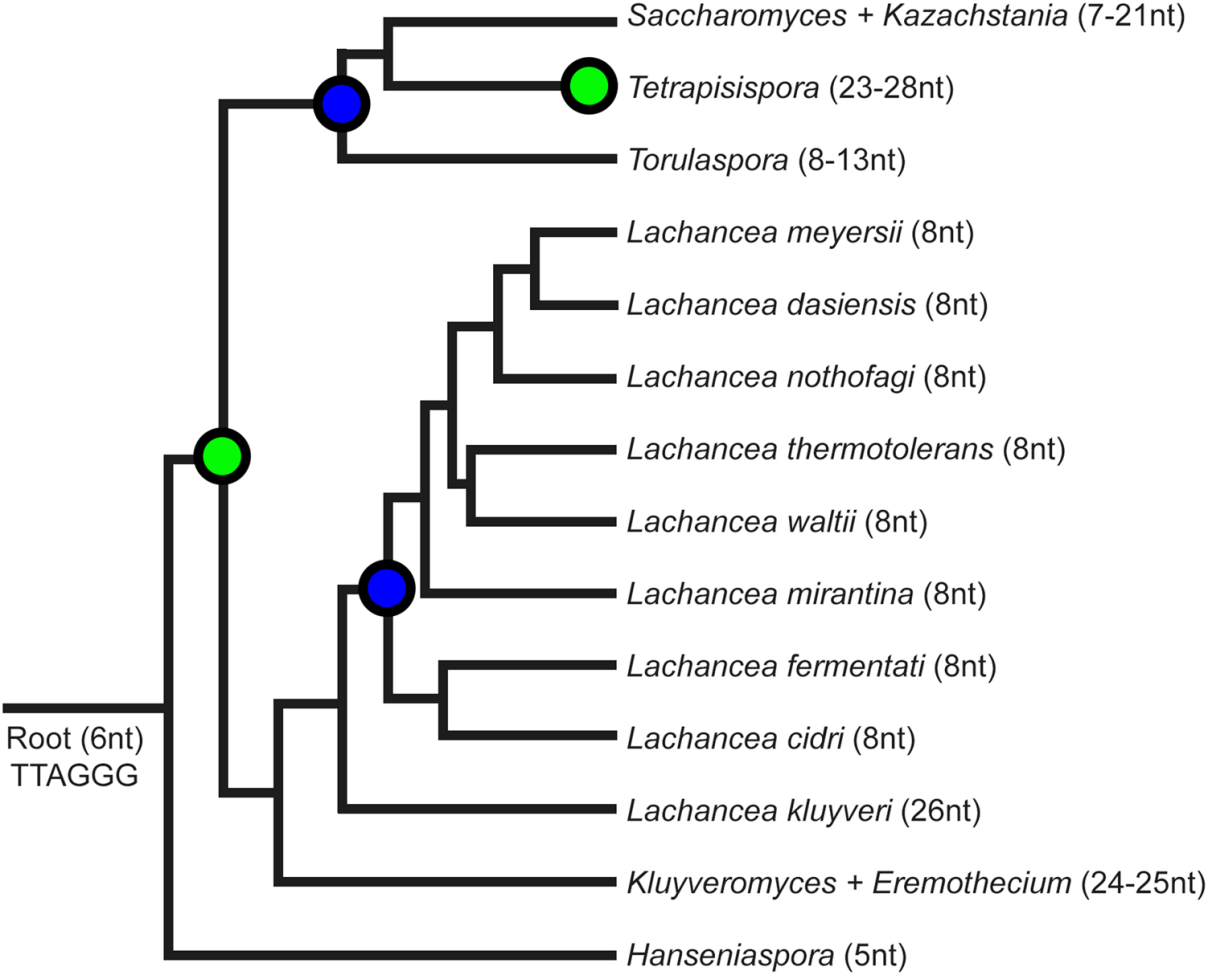
Schematic evolution of telomere motif length in Saccharomycetaceae. The telomere motif length undergoes a series of stretchings (green nodes) and shrinkages (blue nodes). The root represents a common ancestor with Ascomycota and other eukaryotes. Recent publication hypothesizes TTAGGG as an ancestral sequence for all fungi and all eukaryotes, respectively^55^. The scheme of the tree is based on a recent genus-level phylogeny in Saccharomycotina^73^. The motif lengths are based either on results in this work or previous articles^23,74^. The branch lengths in this scheme do not represent evolutionary distances. (Figure created using Adobe Photoshop 22.2.0).

In the genus *Tetrapisispora*, until now, the TR was known only in *T. blattae* (with an unspecified template region) and telomere sequences were found only very recently in three other species of the genus^23^; we also detected the three published telomere candidates, with 25/28, and 25 nt long motifs, respectively, *T. fleetii*,* T. iriomotensis*, and *T. namnaoensis*. Cervenak et al.^23^ also showed that *T. iriomotensis* had telomere motifs 28/29 nucleotides long at the ends of genomic scaffolds. This would make these species record holders in the motif length among Saccharomycetaceae. Except for *T. blattae*, all *Tetrapisispora* analyzed TRs contain direct repeats of template regions with dimeric sub-repeats (ATC)_2_, which probably allow a dual annealing register and the occurrence of the two motifs, 28/25 nt long. *T. fleetii* and *T. namnaoensis* share identical template regions and telomere motifs, while *T. iriomotensis* differs from the previous two by three nucleotide polymorphisms in the telomere sequence (Supplementary Table S2). *T. blattae* has a shorter telomere sequence (25/21 nt) and a TR that does not contain the sub-repeats. The enormous telomere repeat unit size, in fact one of the longest units reported in Saccharomycetaceae, led us to hypothesize that yeast may compensate for a possible low telomerase processivity by expanding the telomere motif to keep the telomere length stable. The hypothetical ancestral telomere sequence TTAGGG^23,55^ is here substituted by motifs of multiple sizes, e.g., by a fourfold increase in *Tetrapisispora*. On the other hand, the genus *Lachancea* exhibits the opposite trend, a shortening of the telomere motif despite the sequence variability in descendant taxa (Fig. 5).

Telomere sequences are relatively well conserved across large groups of higher eukaryotes, where a single telomere type (e.g., TTTAGGG among plants) is the most common and others appear as outliers (e.g. CTCGGTTATGGG in *Allium* sp.)^19,28,56,57^. The apparent extraordinary molecular diversity in yeasts can be related to their short generation times, diversity and early evolutionary divergence: e.g., *Saccharomyces cerevisiae* and *Schizosaccharomyces pombe*, both Ascomycota, are similarly diverse from each other as they are from animals, their ancestors having separated about 420–330 Mya^58^.

Despite the divergence, telomere sequences keep track of guanines (Gs) as one of the most conserved features in all species tested. Certain G-rich sequences form G4 structures involved in gene regulation, telomere protection, and other biological processes^59,60^. It raises questions about the propensity to form G4 as a common telomere sequence feature. G4 formation in vitro was previously confirmed in the telomere repeats S. *pombe* and *S. cerevisiae*^61,62^*.* Biological relevance of G4 in *S. cerevisiae* was also studied in vivo. The telomere G4 stabilization restored compromised capping and was proposed as a rudimentary and possible back-up mechanism in the telomere protection^63^. Thus, we decided to predict if all sequences tested are prone to form G4 and verify this prediction by in vitro measurements. We selected four sequences for the in vitro assays from both predicted extremes and an average range. G4Hunter, QGRS mapper, as well as experimental measurements in vitro (Fig. 4, Supplementary table S4), demonstrated a notable heterogeneity of motifs tested in their propensity to form G4 secondary structures that range from very weak prediction (no G4 in vitro) to very strong ones. The strong prediction was obtained in only five sequences from the list. The best predictions were in *L. mirantina*, *L. thermotolerans*, and *L. waltii*. While the lowest scores were in *Kluyveromyces* sp., the G4 prediction in the real telomere DNA from *S. cerevisiae* is comparable to the scores in hypothetical sequences from only the basic motif (CCACAC)_n_ or to the scores of human telomeres, which is consistent with the previous finding of an accumulation of G4-forming sequences in telomeres of *S. cerevisiae*^24^. Our results imply that the ability to form G4 in vivo may be different in the telomere sequences tested despite the presence of tracks of Gs in all of them.

Another interesting finding is that several telomere-like sequences supported by TR candidates can be found in a single species. Heterogeneity in telomere repeats within a species had been already detected in yeast species^26,64^ and other organisms^30,65,66^. Besides the existence of three error mechanisms proposed to explain intraspecific telomere sequence heterogeneity (telomerase stalling, stuttering and misincorporation)^67^, telomerase RNA paralogs with different template regions were published^28^. These results raise questions about possible pseudogenes of TR candidates, which can complicate the prediction conclusions. If needed, in further experimental progress for selected species, the predictions should be verified by gene deletion or template region modification to conclude for sure that the telomerase RNA is identified.

## Materials and methods

### Genomic data

Datasets were downloaded from public sequence read archives (SRA, NCBI, https://www.ncbi.nlm.nih.gov/sra/). Illumina HiSeq/MiSeq/Illumina Genome Analyzer data from Whole Genome Shotgun (WGS) strategies were preferred for their uniform read length and genome representativeness. Optimal datasets were not available in the case of *Saccharomyces boulardii* (454 GS FLX Titanium), *Kluyveromyces lactis* and *Saccharomyces pastorianus* (HiSeq X Ten), although in general we have avoided HiSeq X Ten as much as possible because this platform systemically under-represents telomere sequences^50^. The read length was correlated with the chances of detecting long tandem repeats, however, for nine species, only 40–50 nt long reads were available. Read length, number of reads used per species and other detailed information can be found in Supplementary Table S1 and S2. TR sequences were downloaded from GenBank (NCBI) according to previously published data^29,33,34,36,44–47^. Template regions were considered, according to coordinates in Waldl et al.^36^. For a complete list of the species analysed and accession numbers, see Supplementary Table S1.

### Tandem repeats finder

The analysis of short tandem repeats was performed using Tandem Repeats Finder (TRFi)^42^ with custom made scripts as described previously^68^, with some modifications, which we call the default setup in this work: the detected motif length was 5–50 nt long and the minimum number of such repeats in tandem was set to five units. This option of five units makes the method stricter than previously used and makes it more selective for reads with lower complexity, less sensitive to sequencing errors at single positions because the consensus is calculated from more repeat units. We omitted prefiltering as the downloaded data exhibited sufficient quality for TRFi analysis. TRFi parameters for the alignment and matching/indel probabilities were set as recommended by default^42^. The vast majority of telomere candidates were actually consensus sequences built from hundreds and thousands of repeat units each. TRFi was repeated with the minimum number of repeats set to two units in three cases (*Kluyveromyces lactis*, *Lachancea kluyveri*, and *Tetrapisispora blattae*). Empirically, the telomere candidates emerged among the first fifty most abundant tandems and only exceptionally was the candidate identified as a much less abundant sequence, or was not identified at all^68^ (Supplementary Table S2). The motifs in the TRFi results are consensual patterns from the detected tandem arrays, possibly containing a certain level of variability and/or degeneracy.

### Prediction of telomere motifs, TRs, and template regions

Novel TRs were predicted in synteny-based homology searches according to a previous study^36^ (Supplementary Table S1). We used tblastx search with a known TR locus + surrounding regions (usually ± 10 Kb) from related organisms as a query to identify a collinear genomic region in the species of interest. Subsequently, a sequence corresponding to the telomerase RNA gene was searched in dot plot analysis and alignment. This workflow provided a very limited number of TR candidates per species, usually only a single possible candidate. Independently, telomere motifs were predicted from TRFi results with respect to the frequency of detected repeats and typical features like unequal distribution of G/C in the complementary strands, presence of two or more adjacent Gs etc. The typical TRFi output was only a short list (in the order of units) of several abundant repeats followed by many others with significantly lower abundance. The telomere candidate was usually one of the abundant repeats, however not exclusively. Then the revision of corresponding telomerase RNA was done as follows: a set of permuted tandems and their reverse complements (always prolonged by one, two etc., up to ten nucleotides from the following unit, labelled as + 1, + 2, up to + 10 in Supplementary Figure S1–S4) were checked using the plain text search (grep—command-line utility in Unix) for their presence either in the whole gene or (if available) between the Ku binding hairpin and Est1 binding site in the telomerase RNA, the positions of which were estimated by local alignment of conserved regions with annotated data in previous work^36^. When the permutation (+ 1 etc.) matched, it was considered as a telomere sequence candidate and the telomerase RNA counterpart as either a result of approach validation or a newly predicted template region in the TR. The final selection of candidates was done manually considering the frequency of the motif and the length of the annealing part. The candidates with very low G/C content (e.g., ATATA in *C. glabrata*) were excluded even if there was a match with overlap + 1 in TR (see Supplementary Table S2). All motifs described above, including selected candidate templates are depicted in Supplementary Figure S1–S4 and marked in Supplementary Table S2. The maximum sequence in the putative TR matching the telomere candidate was then taken as a maximum putative template region. The length estimation of the relative template region was calculated as the ratio between the length of the maximum putative template region and the length of the repeat unit in the predicted telomere sequence (Supplementary Table S1). A pipeline of the procedure used to obtain new telomere motifs and template regions is shown in Fig. 2. Overall, we either: (1) detected the published telomere sequences and template regions when there was congruence between them and our TRFi results, (2) predicted a telomere sequence and a maximum putative template region when these were not available for a given species, but TRFi detected a promising candidate consistent with the region in TR (between Ku and Est1 binding sites if information about them was available), or (3) predicted the telomere sequence, TR, and maximum putative template region for a given species. Only in one case (*K. lactis*) could we not draw any conclusion because of inconsistency between the published TR and the outcome of TRFi results (Supplementary Table S1).

### Prediction of G4 forming sequences in telomere DNAs

Candidate telomere sequences (Supplementary Table S3) were analysed for G4 forming sequences (GFS) using G4Hunter Web Tool^69^ and QGRS mapper^70^. Both tools are used for G4 prediction and count on G-richness and G-skewness of a DNA or RNA sequence. They provided quadruplex propensity scores, full sequences of window peak scores in the case of G4Hunter and peak-sequence scores in QGRS. We simulated telomeres by simple repeats of a candidate unit. In the case of *S. cerevisiae*, we compared GFS in simulated and real sequences. The window size for G4Hunter was set to 25. The analysis by QGRS mapper was performed with default parameters (max length of the GFS = 30, min number of guanidines in G-track = 2, max loop size = 36 nt). In general, the higher the score, the higher the probability of the sequence forming a G4 is. The results of the analyses are presented in (Supplementary Table S3).

### Circular Dichroism (CD) spectroscopy

Synthetic oligonucleotides were purchased from Integrated DNA Technologies (Coralville, Iowa, United States) and diluted in water to a concentration of 100 μM. The oligonucleotides were then heated at 95 °C for 5 min in either 1 mM sodium phosphate buffer (pH 7) and 0.3 mM EDTA or 1 mM sodium phosphate (pH 7), 10 mM potassium phosphate (pH 7) and 90 mM KCl and slowly cooled to room temperature. CD measurements were carried out in a Jasco 815 (Jasco International Co., Ltd., Tokyo, Japan) dichrograph in 1 cm path-length microcells at 23 °C. A set of four scans with a data pitch of 0.5 nm and 200 nm min^−1^ scan speed was averaged for each sample. CD signals were expressed as a difference in the molar absorptions, Δε of the left- and right-handed circularly polarized light^71^.

### Thioflavin T fluorescence assay

Oligonucleotides were further diluted to a 2 μM concentration in 100 mM Tris–HCl (pH 7.5) and 100 mM KCl buffer, heated at 95 °C for 5 min and slowly cooled to room temperature. Thioflavin T (ThT) was diluted in water with addition of KCl to 1 μM final concentration. Experiments were performed in 384-well microplates from CORNING (Flat Bottom Black Polyester). Each condition was tested in triplicate at room temperature. Oligonucleotides and ThT were mixed at 1:0.5 molar ratio to a final volume of 20 μl. Fluorescence emission was collected at 460–700 nm, every 2 nm after excitation at 425 nm in a microplate reader (Spark, Tecan)^72^.

## Supplementary Information

Supplementary Legends.

Supplementary Figure S1.

Supplementary Figure S2.

Supplementary Figure S3.

Supplementary Figure S4.

Supplementary Table S1.

Supplementary Table S2.

Supplementary Table S3.

Supplementary Table S4.
