# Supplementary material for: Extraordinary diversity of telomeres, telomerase RNAs and their template regions in Saccharomycetaceae
Source: Sci Rep. 2021 Jun 17;11:12784. doi: 10.1038/s41598-021-92126-x (PMC8211666; doi:10.1038/s41598-021-92126-x)
Supplement: Supplementary file 1 — Supplementary Legends. [file 41598_2021_92126_MOESM1_ESM.docx]

Supplementary material title page

**Extraordinary diversity of telomeres, telomerase RNAs and their template regions in Saccharomycetaceae**

Vratislav Peska^1*^, Petr Fajkus^1,3^, Michal Bubeník^1,3^, Václav Brázda^1^, Natália Bohálová^1,2^, Jiří Fajkus^1,3^, Sònia Garcia^4*^

^1^Institute of Biophysics, Academy of Sciences of the Czech Republic, Brno CZ–61265, Czech Republic.

^2^Department of Experimental Biology, Faculty of Science, Masaryk University, Brno CZ-62500, Czech Republic

^3^Mendel Centre for Plant Genomics and Proteomics, CEITEC, Masaryk University, Brno CZ-62500, Czech Republic

^4^Institut Botànic de Barcelona (IBB-CSIC, Ajuntament de Barcelona), Passeig del Migdia s/n, Barcelona 08038, Catalonia, Spain.

*Authors for correspondence: [vpeska@ibp.cz](mailto:vpeska@ibp.cz); [soniagarcia@ibb.csic.es](mailto:soniagarcia@ibb.csic.es)

**Supplementary Table S1** Summary of species tested, used datasets, identified telomere, telomerase and template region sequences.

**Supplementary Table S2** Tandem repeats finder output with highlighted telomere sequences and details about datasets.

**Supplementary Table S3** G-quadruplex analysis output.

**Supplementary Table S4** G-quadruplex formation *in vitro* measurements of selected sequences

**Supplementary Figure S1**. Visualisation of all tandem motifs from TRFi output, including candidate template, matching the target region of TRs in *C. glabrata, K. aestuartii, K. dobzhanskii, K. lactis, K. marxianus, L. kluyveri, L. cidri, L. dasiensis, L. fermentati, L. meyersii.*

**Supplementary Figure S2.** Visualisation of all tandem motifs from TRFi output, including candidate template, matching the target region of TRs in *L. mirantina, L. nothofagi, L.* sp. CBS 6924*, L. thermotolerans, L. waltii, S. arboricloa, S. bayanus, S. cerevisiae, S. cerevisiae x kudriavzevii, S. eubayanus.*

**Supplementary Figure S3.** Visualisation of all tandem motifs from TRFi output, including candidate template, matching the target region of TRs in *S. kudriavzevii, S. mikatae, S. paradoxus, S. pastorianus, S. boulardii, S. uvarum, T. blattae, T. fleetii, T. iriomotensis, T. namnaoensis.*

**Supplementary Figure S4.** Visualisation of all tandem motifs from TRFi output, including candidate template, matching the target region of TRs in *T. delbrueckii, T. franciscae, T. maleeae, T. microellipsoides, T. pretoriensis, Z. bailii, Z. rouxii, Z. sapae.*
