# Supplementary figures and images for: Extraordinary diversity of telomeres, telomerase RNAs and their template regions in Saccharomycetaceae
Source: Sci Rep. 2021 Jun 17;11:12784. doi: 10.1038/s41598-021-92126-x (PMC8211666; doi:10.1038/s41598-021-92126-x)

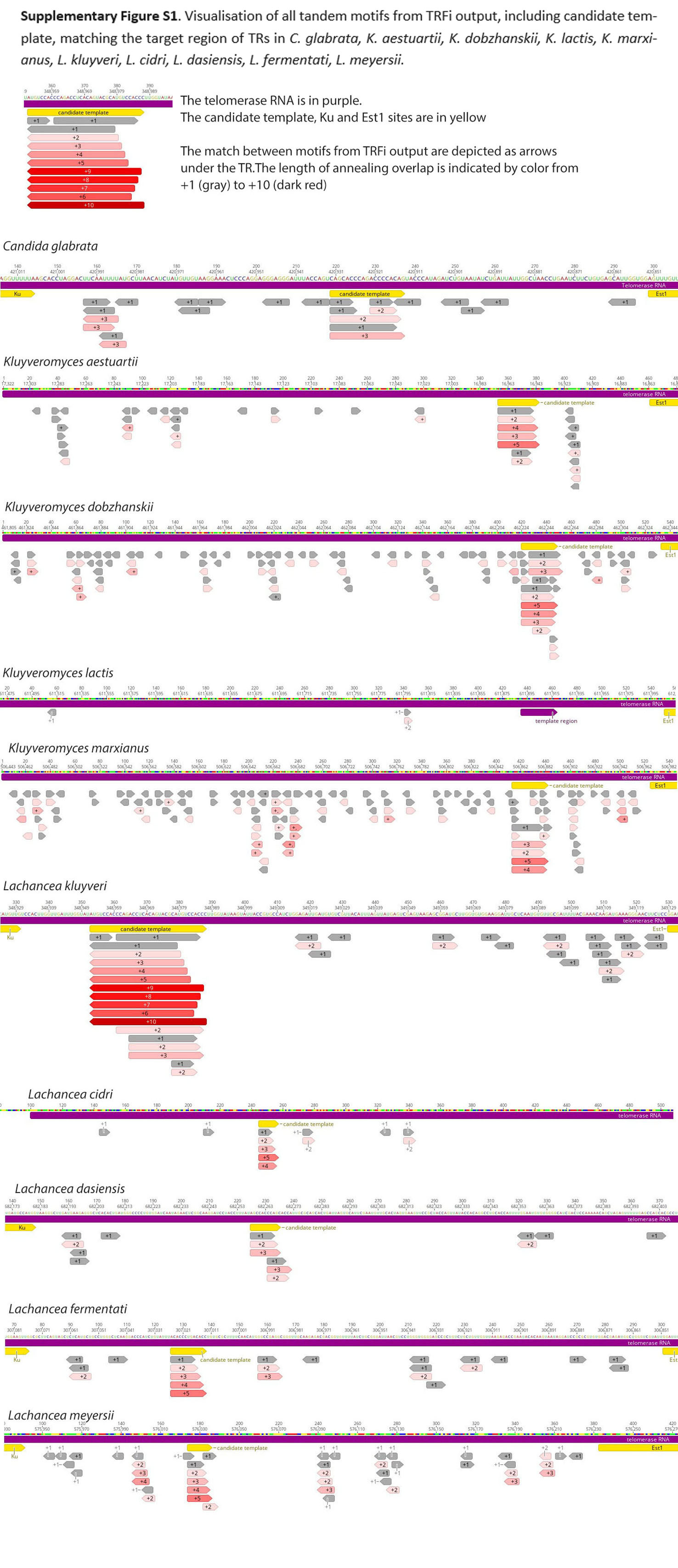

Supplement: Supplementary file 2 — Supplementary Figure S1. [file 41598_2021_92126_MOESM2_ESM.jpg]

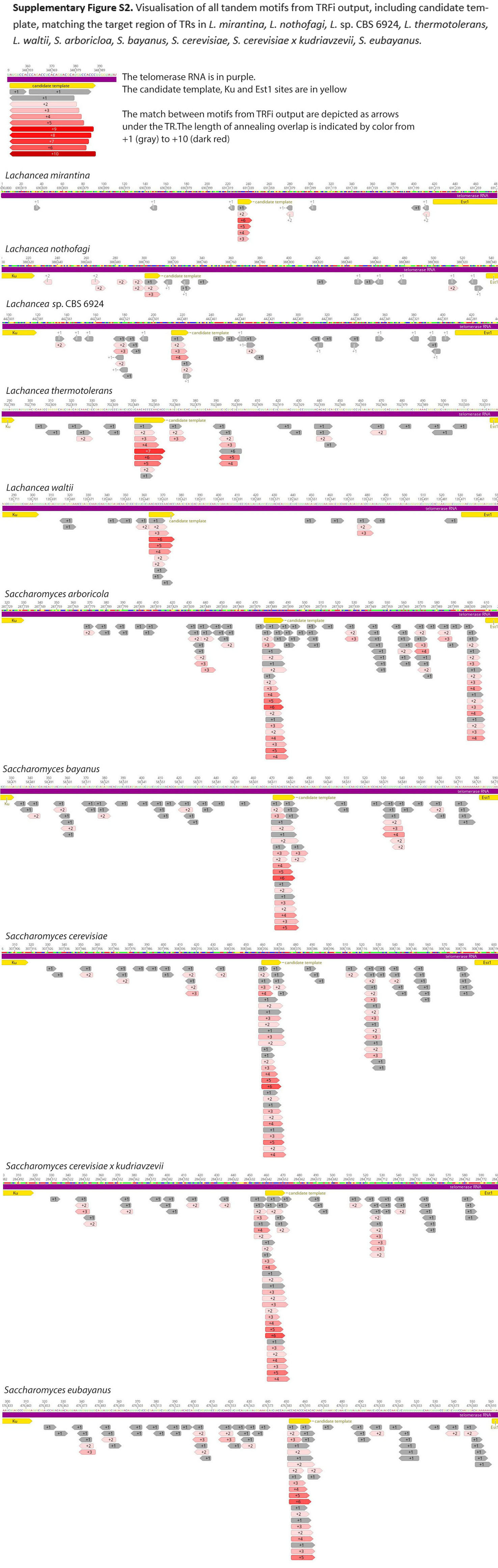

Supplement: Supplementary file 3 — Supplementary Figure S2. [file 41598_2021_92126_MOESM3_ESM.jpg]

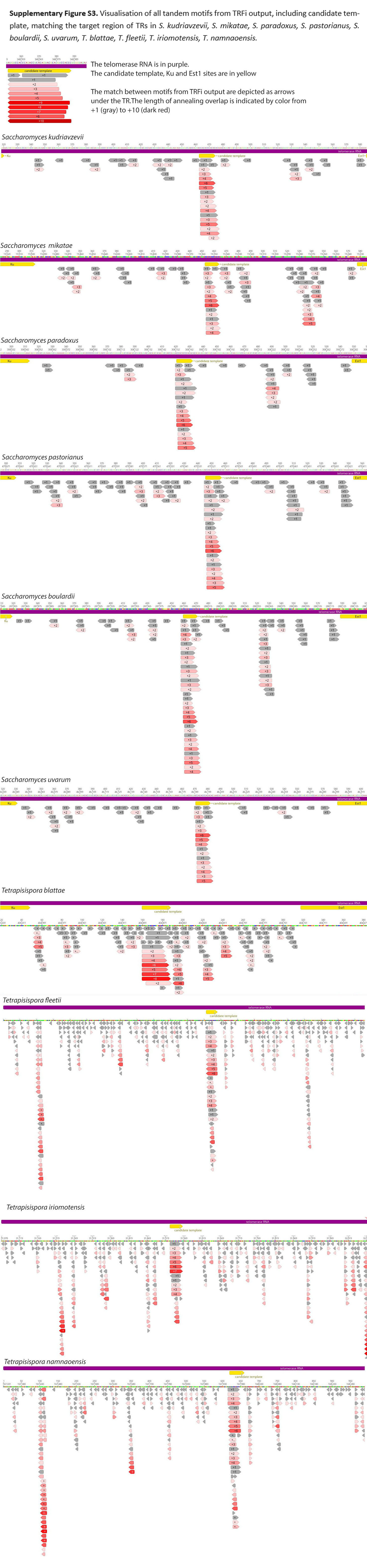

Supplement: Supplementary file 4 — Supplementary Figure S3. [file 41598_2021_92126_MOESM4_ESM.jpg]

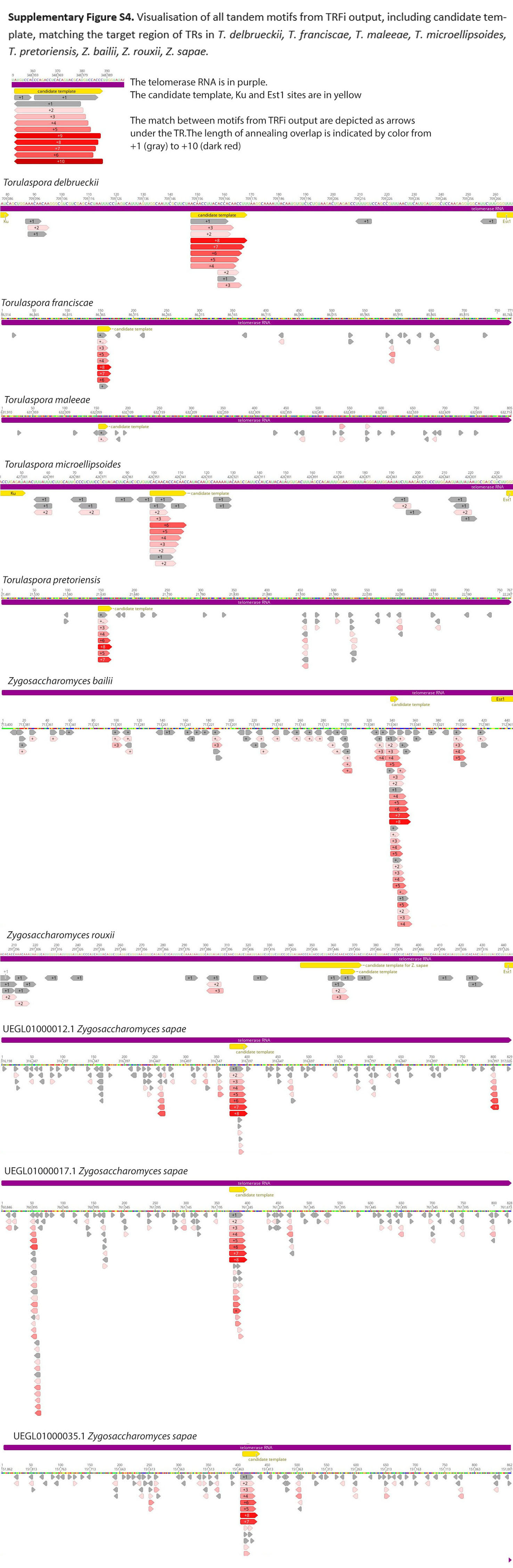

Supplement: Supplementary file 5 — Supplementary Figure S4. [file 41598_2021_92126_MOESM5_ESM.jpg]
